# Supplementary material for: Chromothripsis during telomere crisis is independent of NHEJ, and consistent with a replicative origin
Source: Genome Res. 2019 May;29(5):737–49. doi: 10.1101/gr.240705.118 (PMC6499312; doi:10.1101/gr.240705.118)
Supplement: Supplemental Material [file supp_gr.240705.118_Supplemental_file_1.zip › contigs/annotated_contigs/DB68/contig.2.DB68_length_356_mean_cov_11.8230337079.docx]

**DB68_length_356_mean_cov_11.8230337079**

```
AGGCTGGGCATGGTGGCTCACGCCTGTAATCCCAGGCGTTGGATTTGGGAGGCTGAGGCGGGTGGATCACCTGAAGTCAGGAGTTCAAG
 >chr3:183190822-183191092 - E=2e-148
ACCAGCCTGGCCAACATGGTGAAACCCCTTCTCTACTAAAAATACAAAAATTAGCCAGGCATGATGGTGGTGCCTGTAATCCAAGCTAC

TTGGGAGGCTGAGGCAGGAGAATTGCTTGAACCCAGGGGGTGAAAGTTGCAGTCAGCCGAGATCGCGCCACTGCACTCCAGCCTGGGCA

ACA|GAGCAAGACTCTGCC|TCAAAAAAAAAAAGAAGAAGAAGAAGAAGAAAACAAAAAA|GAGCAGAACTGAGTCATTGTGATGAGAC
                   >chr5:79612791-79612831 - E=1e-09
TCT
```
